# Health inequities as measured by the EQ-5D-5L during COVID-19: Results from New York in healthy and diseased persons

**DOI:** 10.1371/journal.pone.0272252

**Published:** 2022-07-28

**Authors:** Erica I. Lubetkin, Di Long, Juanita A. Haagsma, Mathieu F. Janssen, Gouke J. Bonsel

**Affiliations:** 1 Department of Community Health and Social Medicine, CUNY School of Medicine, New York, New York, United States of America; 2 Department of Public Health, Erasmus MC University Medical Center Rotterdam, Rotterdam, The Netherlands; 3 Section Medical Psychology and Psychotherapy, Department of Psychiatry, Erasmus MC, Rotterdam, The Netherlands; 4 EuroQol Research Foundation, Rotterdam, The Netherlands; Universitat de Valencia, SPAIN

## Abstract

**Introduction:**

The effects of the COVID-19 pandemic caused considerable psychological and physical effects in healthy and diseased New Yorkers aside from the effects in those who were infected. We investigated the relationship between known risk-enhancing and health-promoting factors (social and medical), comorbidity indicators, and, as the primary outcome, health-related quality of life (HRQoL).

**Methods:**

Between April 22 and May 5, 2020, a market research agency (Dynata) administered a digital survey including the EQ-5D-5L and items related to individual characteristics, social position, occupational and insurance status, living situation, exposures (smoking and COVID-19), detailed chronic conditions, and experienced access to care to an existing internet panel representative of New Yorkers.

**Results:**

2684 persons completed the questionnaire. The median age was 48 years old, and most respondents were non-Hispanic white (74%) and reported at least higher vocational training or a university education (83%). During COVID-19, mean HRQoL scores were 0.82 for the EQ-5D-5L index and 79.3 for the EQ VAS. Scores varied for healthy and diseased respondents differently by the above determinants. Lower age, impaired occupational status, loss of health insurance, and limited access to care exerted more influence on EQ-5D-5L scores of diseased persons compared to healthy persons. Among diseased persons, the number of chronic conditions and limited access to health care had the strongest association with EQ-5D-5L scores. While EQ-5D-5L scores improved with increasing age, gender had no noticeable effect. Deprivation factors showed moderate effects, which largely disappeared in (stratified) multivariable analysis, suggesting mediation through excess chronic morbidity and poor healthcare access. Generally, modifying effects were larger in the EQ-5D-5L as compared to the EQ VAS.

**Conclusions:**

Almost all factors relating to a disadvantaged position showed a negative association with HRQoL. In diseased respondents, pre-existing chronic comorbidity and experienced access to health care are key factors.

## Introduction

As COVID-19 spread throughout the United States (U.S.), New York emerged as the Nation’s epicenter of the pandemic, initially having both the highest number of cases and deaths [1]. By April 2020, the COVID-19 incidence rate in New York City and New York was 497 and 293 per 100,000, respectively which was more than five times higher compared to the total U.S. [2]. The Government responded with highly stringent policy and public health measures to control the spread of COVID-19 in March and April 2020, including the closure of schools and non-essential businesses. While the impact of COVID-19, in terms of positive tests, hospitalizations, and deaths, on patients has been widely examined in the population, the impact on the United States general population has received less attention [3–5]. As a result of governmental public health and policy responses at the national and state levels, which include containment and closure policies, economic policies, health system policies and vaccine policies [6, 7], COVID-19 may be associated with psychological, social, and economic effects, and these effects may differ among subgroups based on determinants of health and the presence of chronic conditions [5]. Persons with chronic conditions are at higher risk for COVID-19 infection and adverse outcomes, including more serious illness and mortality [8]. In addition, frequency of foregone and delayed medical care was high during the first wave of the COVID-19 pandemic, which may have led to worsening of symptoms of chronic disease [9].

Since COVID-19 will continue to affect the U.S., perhaps for years to come, public health practitioners and policy makers should have the capacity to measure and track morbidity over time. In order to ensure both a preventive and supportive focus, surveillance should be implemented for both affected and unaffected persons as well as persons with and without chronic conditions. Understanding morbidity, including health-related quality of life (HRQoL), in addition to mortality enables the burden of disease due to COVID-19 to be calculated [10]. HRQoL reflects “how well a person functions in their life and his or her perceived wellbeing in physical, mental, and social domains of health” [11]. Over the past two decades, investigators increasingly have used one measure of HRQoL in general populations throughout the world: the EQ-5D [12, 13]. While investigators routinely have examined scores according to age, gender, and educational attainment [12, 13], the roles of other determinants of health, such as race/ethnicity and income have been examined less frequently. Furthermore, the relationship between factors of specific relevance to COVID-19’s impact (even among those not infected), such as being an essential worker, loss of a job and/or health insurance, perceptions of preparedness for a disaster, and experienced access to care, tend not to be assessed. A study that investigated HRQoL during the COVID-19 pandemic in the US population and its determinants showed that age, income, race/ethnicity, marital status, having a chronic disease and COVID-19 were associated with HRQoL [14]. However, determinants including being an essential worker, loss of a job and/or health insurance, perceptions of preparedness for a disaster, and experienced access to care were not included in this study [14]. Nevertheless, these factors may contribute to health inequalities. For example, being an essential worker, and working in lower paying jobs with minimal protection, if any, are associated with a greater risk of exposure to COVID-19 [15–17]. Additionally, lacking health insurance due to job loss has been associated with poor medication adherence or delayed COVID-19 treatment [18, 19]. Furthermore, population subgroups that vary with regard to race/ethnicity, income, and/or education, might differ with regard to level of general preparedness for a natural disaster such as a pandemic [20]. In a diverse sample of adults with chronic health conditions, Wolf and colleagues found that nearly one in three believed that they were only a little or not at all prepared for a COVID-19 outbreak, and only one in five believed that they were very prepared [21].

Although, to date, no published studies have examined HRQoL in New York during the COVID-19 pandemic, the effect of COVID-19 on mental health has been tracked at the state level over time [22]. Beginning on April 23, 2020, the National Center for Health Statistics and Census Bureau administered the Household Pulse Survey. The Household Pulse Survey is a weekly survey that collects information on the impact of COVID-19 on food security, health status, housing security, educational disruption, employment and mental health among a representative sample of more than 800,000 adult persons from the U.S. Between April 23 and May 5, 2020, New York State ranked first for depressive symptoms and second for anxiety symptoms [22].

The following investigation examines the HRQoL impact of the pandemic amongst residents of New York during a time that New York was the epicenter of the COVID-19 pandemic. We studied the effects for persons with and without pre-existing diseases separately, and explored the modifying role of age, gender, education and social position, employment, health insurance status, living situation, health risks such as smoking, and perceived access to care. The per-protocol hypotheses are as follows:

1. Respondents with a higher social position (based on race/ethnicity, income, and education) have better HRQoL as compared to respondents with a lower social position;
2. Respondents reporting more favourable employment and health insurance status and living situation (being employed, a nonessential worker, having health insurance, living alone, and/or having a higher level of disaster preparedness) have better HRQoL as compared to respondents in a less favourable situation;
3. Respondents who are non-smokers, have no chronic conditions, or report more positive access with their last health care visit, will have better HRQoL as compared to respondents who do not report these factors;
4. Respondents who are healthy will show a different relationship between the above determinants and HRQoL as compared to diseased respondents.

## Methods

### Study design

This study is a web-based cross-sectional study among a general population sample from New York (City and State). Ethical approval for this study was obtained from the Erasmus MC ethics review board (approval MEC-2020-0266). Respondents were recruited by a market research agency (Dynata) that distributed and launched the questionnaires. Study participants were members of the market research agency’s existing voluntary panels and had provided written informed consent to participate in online surveys upon registration. An existing large Internet panel was used with samples designed to be representative of the New York City and New York State (excluding New York City) populations for persons aged 18 to 75 with regard to age, gender, and level of education. Surveys were administered from April 22 to May 5, 2020. At the time that these study data were collected, the New York internet panel consisted of over 3000 persons. Respondents were recruited until the pre-defined quotas for age, gender, and education had been achieved. After completing the survey, participants received an incentive in the form of cash or points.

#### Modifying factors

The survey covered sociodemographic information, including area of residence (zip code), age, gender, race/ethnicity, household annual income level, highest level of education achieved, occupational status, essential worker status (yes/no), job loss as a result of COVID-19 (yes/no), health insurance, loss of insurance as a result of COVID-19 (yes/no), living situation, and smoking status. Twelve self-reported chronic conditions, which included respiratory diseases, heart disease, previous stroke, diabetes mellitus, hernia, (rheumatoid) arthritis, cancer and an open text field for any other conditions, were included as well as an item related to self-reported COVID-19 disease or exposure status. Having no chronic conditions and symptoms or exposure to COVID-19 was defined as ’healthy respondents,’ whereas all other respondents were defined as ‘diseased.’ Household annual income was categorized into six groups while education was categorized into three groups. The International Standard Classification of Education (ISCED) was used to categorise educational level into low (primary school, lower secondary school or lower vocational training), middle (intermediate and higher secondary school, or intermediate vocational training, and high (higher vocational training or university education) [23]. Essential workers are defined as those persons who conduct operations or services in industries that are considered to be essential to ensure the continuity of critical functions in the U.S.

Disaster preparedness items were from an optional module of the Behavioral Risk Factor Surveillance System (BRFSS) [24]. The BRFSS is an annual surveillance survey that is administered by the Centers for Disease Control and Prevention (CDC). The BRFSS collects data by telephone in all 50 states, at state level, as well as the District of Columbia and three US territories and aims to monitor modifiable risk factors contributing to the leading causes of morbidity and mortality in the population. The responses on the items were added, and total scores ranged from 8 to 24. These responses were categorized into five levels: not prepared (total score 8–11), somewhat not prepared (12–14), somewhat prepared (15–17), somewhat well-prepared (18–20) and well-prepared (21–24).

### Primary health outcome measures

The survey included the EQ-5D-5L that includes five dimensions: mobility, self-care, usual activities, pain/discomfort, and anxiety/depression. Each dimension has five ordered response categories: no problems, slight problems, moderate problems, severe problems, and extreme/unable to. The EQ-5D-5L level sum score (LSS) is an equally weighted score calculated by summarizing the score on each of the five dimensions of EQ-5D-5L. The EQ-5D-5L level sum score ranges from 5 (best; if all domains have level 1) to 25 (worst; all domains have level 5). The EQ-5D-5L index was calculated using the recently published specific value set for U.S., and ranges from below 0 (worse than death) to 1 (best health) [25]. The EQ-5D measure also consists of a visual analogue scale (EQ VAS) for general health that ranges from 0 (worst imaginable health) to 100 (best imaginable health).

### Data analysis

A detailed analysis of non-responders was not possible due to the system of recruitment used. For the analysis of dropouts, specifically to compare the difference in the distribution in risk factors between dropouts and completers in the study, analysis of variance (ANOVA) was used for continuous variables and Chi-square tests for categorical variables. Descriptive statistics assessed the sample characteristics by EQ outcomes, in addition to preparing determinant selection for regressions. Univariable and multivariable linear regression analysis estimated the association between the EQ outcomes and health determinants. Non-significant terms in the model were not excluded as they are of interest to our study. Likelihood ratio test was assessed for statistical significance. The significance level was set at 0.05. All analyses were carried out using R 3.6.3 [26].

## Results

### Sample

Three thousand forty eight persons agreed to participate. Of the 2684 (88%) respondents completing the questionnaire, 2657 (99%) respondents were included in our analysis. Compared to respondents, dropouts who did not complete the survey (n = 364) were significantly younger and mostly female (S1 Table).

Of the respondents 1045 (39%) were living in New York City and 1612 (61%) in New York State, excluding New York City. Respondents had a mean age of 47.4 years (SD 15.5) and females comprised 55.1% of the sample (Table 1). The majority were non-Hispanic white (73.5%) and had a high educational level (82.6%). Job loss due to COVID-19 was frequently reported (24%). More than 30% of respondents were essential workers. Almost 20% of respondents reported that they (may) have been exposed to or infected by COVID-19. More than 44% of respondents reported having one or more chronic diseases.

**Table 1:** Characteristics of respondents.

Determinants of health	Characteristics	Frequency (%) (N = 2657)
	**Age**	
	Median (IQR)	48.0 (26.0)
	**Age groups**	
	18–24 yrs.	188 (7.1%)
	25–34 yrs.	465 (17.5%)
	35–44 yrs.	527 (19.8%)
	45–54 yrs.	527 (19.8%)
	55–64 yrs.	472 (17.8%)
	65–75 yrs.	478 (18.0%)
	**Gender**	
	Male	1193 (44.9%)
	Female	1464 (55.1%)
***Social position***	**Race/ethnicity**	
	Non-Hispanic White	1953 (73.5%)
	Non-Hispanic Black	192 (7.2%)
	Hispanic	319 (12.0%)
	Non-Hispanic Asian	193 (7.3%)
	**Level of education** 1	
	High	2196 (82.6%)
	Middle	359 (13.5%)
	Low	102 (3.8%)
	**Household income**	
	Q5–richest (≥150,000$)	472 (17.8%)
	Q4–rich (100,000–149,999$)	509 (19.2%)
	Q3–middle (75,000–99,999$)	398 (15.0%)
	Q2–poor (50,000–74,999$)	436 (16.4%)
	Q1–poorest (≤49,999$)	652 (24.5%)
	Unwilling to tell	190 (7.2%)
	**Neighborhood/residency**	
	Manhattan	386 (14.5%)
	Staten Island	42 (1.6%)
	Bronx	102 (3.8%)
	Brooklyn	208 (7.8%)
	Queens	307 (11.6%)
	NY State (other than NYC)	1612 (60.7%)
***Employment, living situation, and health insurance***	**Occupational status**	
	Employed	1448 (54.5%)
	Unemployed^1^	525 (19.8%)
	Retired	525 (19.8%)
	Unable to work	159 (6.0%)
	**Job loss in household due to COVID-19**	
	No	2019 (76.0%)
	Yes	638 (24.0%)
	**Essential worker status**	
	Not essential worker^3^	1795 (67.6%)
	Essential worker: male	472 (17.8%)
	Essential worker: female	390 (14.7%)
	**Living situation**	
	Living alone	605 (22.8%)
	Living with partner and/or family	1950 (73.4%)
	Other	102 (3.8%)
	**Health insurance**	
	Yes	2429 (91.4%)
	No	107 (4.0%)
	Unknown	121 (4.6%)
	**Loss of health insurance due to COVID-19**	
	No	2387 (89.8%)
	Yes	270 (10.2%)
	**Disaster preparedness**	
	Well prepared (total score 8–11)	1161 (43.7%)
	Somewhat well prepared (total score 12–14)	828 (31.2%)
	Somewhat prepared (total score 15–17)	422 (15.9%)
	Somewhat not prepared (total score 18–20)	163 (6.1%)
	Not prepared (total score 21–24)	83 (3.1%)
***Exposures and conditions***	**Smoking status (including e-cigarettes)**	
	Not at all	1838 (69.2%)
	Some days	198 (7.5%)
	Every day	321 (12.1%)
	**Self-reported COVID-19 status**	
	Not infected	2138 (80.5%)
	May be infected	439 (16.5%)
	Infected but recovered	39 (1.5%)
	Infected and not recovered	41 (1.5%)
	**Number of chronic conditions**	
	0	1481 (55.7%)
	1	753 (28.3%)
	2	265 (10.0%)
	3	94 (3.5%)
	4 and more	64 (2.4%)
***Health care access***	**Expected access to health care**	
	Expect no difficulties to go	987 (37.1%)
	Expect difficulties to go	999 (37.6%)
	Don’t expect to go because I’m afraid of COVID-19	432 (16.3%)
	Don’t expect to go because I will not qualify to get appointments	239 (9.0%)
	**Last visit to health care (outpatient)**	
	More than 3 months ago	1533 (57.7%)
	1 to 4 weeks ago	277 (10.4%)
	About 1 to 3 months ago	700 (26.3%)
	Last week	147 (5.5%)
	**Recall last healthcare visit, experience with access**	
	Very good/Always good	1301 (49.0%)
	Good/Usually good	961 (36.2%)
	Fair/Sometimes good	325 (12.2%)
	Bad/Usually not good	50 (1.9%)
	Very bad/Never good	20 (0.8%)

^1^ Education categories: High (ISCED 5 and above), Middle (ISCED 3–4), Low (ISCED 0–2).

^2^ Including caregiver and student

^3^ Non-essential worker status category includes also retired, unemployed, students and unable to work.

### HRQoL

For all respondents, mean (SD) EQ-5D-5L index, LSS, and EQ VAS were 0.82(0.26), 7.5(3.4), and 79.3(17.4), respectively. Mean EQ outcome scores varied by levels of response in each characteristic category (Table 2). Almost every factor relating to a more disadvantaged position showed a worse score in HRQoL, except for age. Scores of HRQoL improved with higher age category. Non-Hispanic whites and non-Hispanic Asians had the best score in HRQoL compared to other racial/ethnic groups. Middle income groups had better scores in HRQoL compared to the other income groups.

**Table 2:** Mean (SD) EQ-5D-5L index, level sum score, and EQ VAS scores by respondent’s characteristics.

	EQ-5D-5L index	EQ-5D-5L level sum score	EQ VAS
*Characteristics*	*Mean (SD)*	*Mean (SD)*	*Mean (SD)*
***Total***	0.82 (0.26)	7.5 (3.4)	79.3 (17.4)
***Age groups***			
18–24 yrs.	0.67 (0.33)	9.7 (4.3)	77.2 (21.0)
25–34 yrs.	0.77 (0.30)	8.2 (3.9)	78.2 (19.2)
35–44 yrs.	0.80 (0.29)	7.8 (3.8)	79.3 (18.1)
45–54 yrs.	0.84 (0.25)	7.3 (3.2)	79.0 (16.5)
55–64 yrs.	0.87 (0.21)	6.9 (2.7)	79.7 (16.2)
65–75 yrs.	0.89 (0.16)	6.7 (2.2)	81.2 (15.1)
***Gender***			
Male	0.82 (0.28)	7.6 (3.7)	79.4 (16.9)
Female	0.82 (0.24)	7.5 (3.2)	79.3 (17.8)
***Race/ethnicity***			
Non-Hispanic White	0.84 (0.24)	7.3 (3.2)	80.6 (16.0)
Non-Hispanic Black	0.76 (0.31)	8.3 (3.8)	76.5 (18.5)
Hispanic	0.72 (0.32)	9.0 (4.2)	72.7 (23.3)
Non-Hispanic Asian	0.87 (0.22)	6.9 (3.0)	80.2 (15.9)
***Level of education*** 1			
High	0.83 (0.26)	7.5 (3.4)	80.2 (16.4)
Middle	0.83 (0.22)	7.4 (2.9)	77.4 (18.8)
Low	0.66 (0.41)	9.6 (5.1)	67.4 (26.8)
***Household income***			
Q5–richest (≥150,000$)	0.82 (0.29)	7.5 (3.8)	82.4 (15.1)
Q4–rich (100,000–149,999$)	0.85 (0.24)	7.2 (3.2)	82.7 (14.9)
Q3–middle (75,000–99,999$)	0.85 (0.23)	7.3 (3.0)	79.8 (15.0)
Q2–poor (50,000–74,999$)	0.84 (0.21)	7.4 (2.8)	79.1 (16.5)
Q1–poorest (≤49,999$)	0.76 (0.30)	8.3 (3.9)	73.8 (20.9)
Unwilling to tell	0.88 (0.21)	6.8 (2.8)	81.2 (18.1)
***Neighborhood/residency***			
Manhattan	0.78 (0.30)	8.1 (3.9)	80.6 (18.4)
Staten Island	0.83 (0.26)	7.3 (3.4)	78.7 (18.1)
Bronx	0.74 (0.30)	8.7 (4.0)	75.2 (22.5)
Brooklyn	0.81 (0.29)	7.8 (3.8)	77.8 (17.5)
Queens	0.83 (0.25)	7.4 (3.3)	78.8 (17.7)
NY State	0.84 (0.25)	7.4 (3.2)	79.6 (16.7)
***Occupational status***			
Employed	0.85 (0.24)	7.1 (3.1)	81.5 (15.8)
Unemployed^2^	0.73 (0.31)	8.8 (4.0)	75.9 (18.7)
Retired	0.89 (0.17)	6.7 (2.3)	80.4 (16.5)
Unable to work	0.62 (0.39)	10.0 (4.9)	67.8 (23.1)
***Job loss in household due to COVID-19***			
No	0.85 (0.24)	7.2 (3.1)	79.8 (16.7)
Yes	0.74 (0.32)	8.6 (4.1)	77.7 (19.4)
***Essential worker status***			
Not essential worker	0.84 (0.23)	7.2 (3.1)	79.2 (16.9)
Essential worker	0.78 (0.31)	8.2 (4.0)	79.7 (18.5)
***Living situation***			
Living alone	0.83 (0.27)	7.3 (3.4)	78.2 (18.7)
Living with partner and/or family	0.82 (0.25)	7.5 (3.3)	79.9 (16.7)
Other	0.73 (0.38)	8.7 (4.9)	74.9 (20.8)
***Health insurance***			
Yes	0.83 (0.25)	7.4 (3.2)	79.9 (16.7)
No	0.70 (0.38)	9.1 (4.8)	74.7 (20.7)
Unknown	0.71 (0.39)	8.9 (4.9)	72.8 (24.2)
***Loss of health insurance due to COVID-19***			
No	0.85 (0.23)	7.2 (3.0)	79.9 (16.7)
Yes	0.59 (0.39)	10.7 (4.9)	73.9 (22.2)
***Disaster preparedness***			
Well prepared	0.84 (0.27)	7.2 (3.5)	82.7 (16.0)
Somewhat well prepared	0.86 (0.18)	7.1 (2.4)	79.6 (15.7)
Somewhat prepared	0.77 (0.30)	8.3 (3.9)	74.4 (19.3)
Somewhat not prepared	0.70 (0.30)	9.1 (3.9)	72.2 (18.8)
Not prepared	0.66 (0.40)	9.8 (5.3)	69.0 (24.3)
***Smoking status (incl. e-cigarettes)***			
Not at all	0.87 (0.21)	6.9 (2.8)	80.5 (16.4)
Some days	0.70 (0.30)	9.3 (3.9)	75.3 (21.3)
Every day	0.72 (0.34)	8.9 (4.4)	77.3 (18.2)
***COVID-19 status***			
Not infected	0.87 (0.19)	6.8 (2.6)	81.0 (16.1)
May be infected	0.66 (0.30)	9.9 (4.0)	71.7 (20.4)
Infected but recovered	0.55 (0.45)	10.9 (5.5)	74.9 (23.7)
Infected and not recovered	0.11 (0.58)	16.1 (7.2)	79.9 (19.6)
***Number of chronic conditions***			
0	0.91 (0.18)	6.4 (2.5)	84.2 (13.9)
1	0.76 (0.29)	8.5 (3.8)	75.1 (18.5)
2	0.70 (0.29)	9.3 (3.6)	72.3 (19.2)
3	0.61 (0.33)	10.2 (3.8)	67.6 (22.0)
4 and more	0.43 (0.36)	12.5 (4.1)	62.9 (19.2)
***Expected access to health care***			
Expect no difficulties to go	0.82 (0.29)	7.6 (3.7)	81.7 (16.6)
Expect difficulties to go	0.83 (0.23)	7.5 (3.1)	78.4 (16.6)
Don’t expect to go because I’m afraid of COVID-19	0.81 (0.26)	7.6 (3.3)	76.0 (19.1)
Don’t expect to go because I will not qualify to get appointments	0.83 (0.29)	7.4 (3.9)	79.3 (19.4)
***Recall last healthcare visit, experience with access***			
Very good/Always good	0.86 (0.24)	7.0 (3.1)	83.1 (16.0)
Good/Usually good	0.82 (0.23)	7.6 (3.1)	78.2 (16.2)
Fair/Sometimes good	0.72 (0.31)	9.1 (4.2)	70.2 (19.8)
Bad/Usually not good	0.57 (0.39)	10.9 (4.9)	66.8 (20.9)
Very bad/Never good	0.49 (0.52)	11.6 (6.4)	67.5 (26.1)

^1^ Education categories: High (ISCED 5 and above), Middle (ISCED 3–4), Low (ISCED 0–2).

^2^ Including caregiver and student

In terms of EQ-5D-5L index and LSS, the respondents with no chronic conditions and age 65–75 had the best health (EQ-5D-5L index mean (SD) 0.91(0.18), 0.89(0.16), and LSS 6.4(2.5), 6.7(2.2), respectively). Respondents who reported exposure to, or infection with, COVID-19 and respondents with four or more chronic conditions had the worst scores (EQ-5D-5L index mean (SD) 0.11(0.58) and 0.43(0.36); and LSS 16.1(7.2), and 12.5(4.1), respectively). Respondents with no chronic conditions and a very good experience with access to health care had the highest EQ VAS scores (mean (SD) 84.2 (13.9), and 83.1(16.0), respectively). Respondents with four or more chronic conditions and very bad access to health care had the worse VAS scores (mean (SD) 62.9(19.2) and 67.5(26.1), respectively). Compared to the other groups, respondents not infected with COVID-19 had the highest VAS scores.

**Fig 1:**
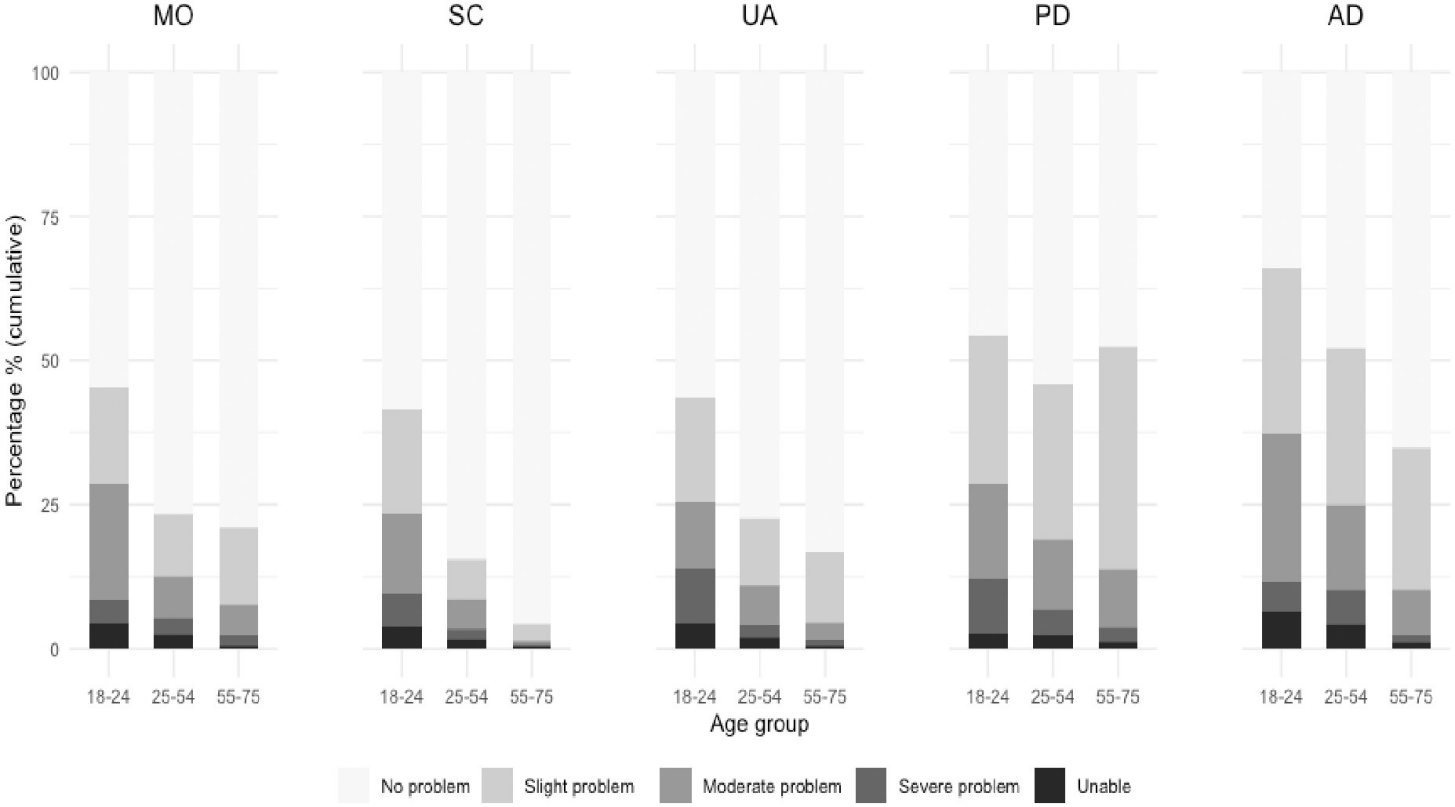
Distribution of EQ-5D-5L dimensions by age groups^1^. 1 MO, SC, UA, PD, and AD are short for the EQ-5D-5L dimensions: mobility, self-care, usual activity, pain/discomfort, anxiety/depression.

Fig 1 presents the distribution of the five dimensions of the EQ-5D-5L according to three age groups. Overall, more respondents reported some problems in the dimension pain/discomfort and anxiety/depression than in any of the other three dimensions. A steep gradient was found in each dimension between age groups, except for pain/discomfort. Older people had a higher share of “no problems” in each dimension, except for pain/discomfort where “slight problems” was more prevalent.

#### Regression analysis

Table 3 reports the results of the exploratory analysis of the modifying role of determinants for the three EQ outcomes (EQ-5D-5L index, LSS, and EQ VAS) and healthy and diseased respondents separately. The EQ-5D-5L and LSS were linearly rescaled to range from 0 to 100 for easy comparison of the regression coefficients. The results with the EQ-5D-5L index serve as the point of departure. In the univariable analysis, almost all factors relating to a disadvantaged position showed the expected negative association with EQ-5D-5L index scores, except age, where being older benefitted HRQoL. Additionally, being non-Hispanic Asian, middle-educated, retired, or having a middle income was ’better.’ People with chronic conditions and (very) bad access to health care had the most negative (‘poorest’) coefficients.

**Table 3:** EQ-5D-5L index, level sum score, and EQ VAS, univariable and multivariable analysis in healthy and diseased respondents ^1^,^2^.

	Univariable analysis N = 2657			Multivariable analysis for Healthy respondents N = 2657			Multivariable for Diseased respondents N = 2657		
	Scaled index^3^	Rescaled level sum score^4^	VAS^5^	Scaled index^3^	Rescaled level sum score^4^	VAS^5^	Scaled index^3^	Rescaled level sum score^3^	VAS^5^
	Beta	Beta	Beta	Beta	Beta	Beta	Beta	Beta	Beta
**Intercept**	/	/	/	95.0	96.1	89.8	91.5	93.8	91.7
**Age group**	*	*					*	*	
25–34 yrs.	10.4	7.5	1.0	3.2	2.5	-3.7	4.8	3.7	-1.3
35–44 yrs.	13.8	9.5	2.1	3.8	2.6	-3.9	7.0	5.0	-3.6
45–54 yrs.	17.4	12.2	1.8	2.2	1.8	-4.9	9.2	6.4	-3.4
55–64 yrs.	20.2	13.9	2.5	3.0	2.1	-5.0	9.6	6.8	-4.5
65–75 yrs.	22.5	15.3	4.0	2.3	1.7	-5.0	12.3	8.4	-1.2
**Gender**									
Female	0.5	0.1	-0.1	/	/	/	/	/	/
**Race/ethnicity**	*	*	*	*	*				*
Non-Hispanic black	-8.2	-5.2	-4.1	-0.2	-0.3	1.4	-1.0	-0.1	-2.7
Hispanic	-12.2	-8.2	-7.9	-2.4	-1.3	-2.8	2.6	1.4	-5.5
Non-Hispanic asian	3.1	2.0	-0.4	4.2	3.0	0.2	5.0	2.8	-0.4
**Level of education**	*	*	*	*	*	*			*
High	-0.6	-0.5	2.7	-0.6	-0.4	2.1	-4.0	-2.7	0.1
Low	-17.7	-11.1	-10.0	-11.6	-7.2	-6.8	-5.9	-3.3	-6.3
**Household income**	*	*	*						*
Q5–Richest	-2.4	-1.2	2.6	0.8	0.7	2.4	-2.1	-1.3	1.7
Q4–Rich	0.3	0.2	2.9	0.3	0.3	1.4	-3.3	-1.7	1.9
Q2–Poor	-0.7	-0.6	-0.7	-0.5	0.1	1.6	-1.4	-1.6	-2.7
Q1–Poorest	-8.5	-5.3	-6.0	-1.3	-0.7	-1.0	-5.1	-3.2	-3.8
Unwilling to tell	3.2	2.4	1.4	-0.2	0.1	1.5	0.7	0.7	0.3
**Residency**	*	*							
NY State	3.4	2.4	0.7	1.3	0.9	0.9	0.0	0.2	-0.7
**Occupational status**	*	*	*			*	*	*	*
Unemployed	-12.3	-8.6	-5.5	-0.5	-0.7	-0.6	-7.0	-4.8	-1.2
Retired	3.2	1.9	-1.1	-0.3	-0.5	1.3	-5.6	-4.1	-5.0
Unable to work	-22.9	-14.6	-13.7	-3.1	-1.9	-4.8	-21.4	-13.9	-5.8
**Job loss due to COVID-19**	*	*	*			*			
Yes	-10.3	-6.9	-2.1	1.6	0.9	1.9	-1.8	-0.9	1.1
**Essential worker status**	*	*					*	*	
Essential worker	-6.9	-4.6	0.5	-0.6	-0.6	1.4	-4.4	-2.9	-0.1
**Living situation**	*	*	*	*	*				
Living with partner and/or family	-1.1	-1.0	1.7	-1.6	-1.1	0.2	-2.0	-1.8	-0.6
Other	-10.4	-6.9	-3.3	-8.3	-5.8	-1.5	-8.5	-5.4	-2.3
**Health insurance**	*	*	*				*	*	
No health insurance	-12.8	-8.2	-5.1	-3.1	-2.4	-0.1	-5.9	-2.6	3.2
Unknown	-12.0	-7.3	-7.1	-1.9	-1.5	-2.7	-11.8	-6.3	-1.3
**Loss of health insurance due to COVID-19**	*	*	*				*	*	*
Yes	-26.1	-17.7	-6.1	-1.7	-0.8	-1.7	-12.5	-8.8	-3.3
**Disaster preparedness**	*	*	*	*	*	*	*	*	*
Somewhat well prepared	-1.7	0.7	-3.0	-1.5	-1.3	-2.3	3.5	1.8	-2.3
Somewhat prepared	- 7.7	-5.6	-8.3	-2.5	-2.2	-3.8	-2.0	-1.8	-5.6
Somewhat not prepared	-14.1	-9.6	-10.4	-0.3	-0.7	-3.2	-5.6	-3.9	-4.6
Not prepared	-18.8	-12.8	-13.7	-6.6	-4.6	-11.6	-8.2	-6.0	-6.0
**Smoking status (including e-cigarettes)**	*	*	*	*	*		*	*	
Some days	-16.7	-11.9	-5.2	-3.7	-3.2	0.0	-7.5	-5.6	-2.7
Every day	-14.9	-9.8	-3.2	-0.7	-0.2	-1.0	-6.3	-4.1	0.0
**Number of chronic conditions**	*	*	*				*	*	*
1	-15.0	-10.5	-9.2	/	/	/	-1.6	-1.3	-3.3
2	-21.3	-14.6	-12.0	/	/	/	-8.9	-6.1	-6.0
3	-29.7	-19.3	-16.7	/	/	/	-14.7	-9.1	-10.9
4 and more	-47.9	-30.9	-21.3	/	/	/	-27.0	-16.9	-12.4
**Expected access to health care**			*						
Expect difficulties to go	1.0	0.1	-3.2	/	/	/	/	/	/
Don’t expect to go because I’m afraid of COVID-19	- 0.3	-0.4	-5.6	/	/	/	/	/	/
Don’t expect to go because I will not qualify to get appointments	1.3	0.6	-2.4	/	/	/	/	/	/
**Recall last healthcare visit, experience with access**	*	*	*	*	*	*	*	*	*
Good/Usually good	- 4.2	-3.1	-4.9	-1.4	-1.2	-3.0	-2.6	-2.1	-4.8
Fair/Sometimes good	-14.9	-10.6	-12.9	-4.9	-3.1	-7.8	-8.6	-6.7	-9.9
Bad/Usually not good	-29.9	-19.9	-16.3	-1.6	-1.3	-2.4	-22.7	-15.1	-12.9
Very bad/Never good	-37.1	-23.0	-15.6	-8.2	-4.2	-15.6	-28.3	-16.8	-5.4

Reference group in each analysis: age 18–24 yrs., male, middle-educated, middle annual household income ($75,000–99,999), resides in New York City, employed, no job loss due to COVID-19, not essential worker, living alone, has health insurance, no insurance loss due to COVID-19, well prepared for disaster, non-smoker, not infected with COVID-19, no chronic conditions, expected access to health care as used to, experience with access is very good/always good.

“*” represents significance (p value<0.05).

For the univariable analysis, intercepts were not shown in the table, therefore beta coefficients of intercepts were marked with ‘/’. For the multivariable analysis, several determinants were not included in specific models (gender, number of chronic conditions, and expected access to health care), and, therefore, the beta coefficients of these determinants were marked with ‘/’.

The outcomes are (re)scaled to a 0 to100 scale to allow for an easy comparison of beta coefficients among outcomes, although this may make the beta coefficients less directly interpretable.

^1^Healthy respondents are those who reported no chronic conditions and not infected with COVID-19

^2^ Diseased respondents are those who reported one or more chronic condition(s) and/nor (possibly) infected with COVID-19

^3^ Scaled index is scaled as 100 times index to allow comparison with the rescaled level sum score and VAS

^4^ Rescaled level sum score is rescaled as 125 times (5 times level sum score) to allow comparison with the scaled index and VAS

^5^ VAS refers to “visual analogue score” to allow comparison with the scaled index and rescaled level sum score

In the multivariable analysis for *healthy* respondents, only some factors showed a significant impact: race/ethnicity, level of education, living situation, disaster preparedness, smoking status, and access to healthcare. Being non-Hispanic Asian was the only factor that had positive (significant) impact. Low-educational attainment, not being prepared for a disaster, and very bad access to healthcare had the most negative impact. In the multivariable analysis for *diseased* respondents, all factors showed a significant impact with the exceptions of race/ethnicity, level of education, household income, residency, and job loss due to COVID-19. Only age and being somewhat well prepared for a disaster had a positive (significant) impact. Respondents who reported being unable to work, had three or more chronic conditions, and who reported (very) bad access to health care had the lowest coefficients.

*EQ VAS*. In the univariable analysis, results were close to that of the EQ-5D-5L index, except that the size of the coefficients was considerably lower with most factors. Worst off were those who were unable to work, were not prepared for a disaster, had three or more chronic conditions, and or had (very) bad access to health care.

The multivariable analysis for *healthy* respondents showed a contributing impact for only a few factors: level of education, occupational status, job loss due to COVID-19, level of disaster preparedness, and experience with access to healthcare. High education and job loss had a positive (significant) impact. Not prepared for disaster and reporting fair to very bad access to healthcare had the worst impact. The multivariable analysis for *diseased* respondents showed distinctly different results with many significant factors. People with three or more chronic conditions and fair or bad access to health care had the lowest coefficients.

## Discussion

This investigation is the first to examine HRQoL for a representative sample of New Yorkers when New York was the epicenter of the COVID-19 pandemic; it also was the first to systematically reveal different patterns of impact on persons with and without (non-COVID-19-related) pre-existing morbidity. Differences in HRQoL are based on numerous factors, including individual characteristics, social position, occupational and health insurance status and living situation, exposures and chronic conditions, and access to healthcare. Generally, the negative impact of the pandemic is amplified in diseased persons, as data suggest in part through limited health care access.

With regard to our first hypothesis, while scores tended to differ between categories of race/ethnicity and income for the univariable analysis, the magnitude of these differences was relatively small as compared to other factors examined for the multivariable analysis. By contrast, other investigators have noted an association between lower income and lower EQ-5D scores in the U.S. general population [27–29]. Perhaps the slightly lower scores amongst the most affluent groups represents the inability to control life circumstances and the reduced social participation due to social distancing measures that had been implemented [30]. The reduction or lack of a difference in EQ-5D scores by race/ethnicity, after adjustment for socioeconomic status, has been previously noted [27, 28, 31]. For education, compared to diseased respondents, healthy respondents in the lowest category of educational attainment had greater impairments in EQ-5D-5L scores, but the same magnitude of impairment was noted for healthy and diseased respondents for the EQ VAS. This indicates that the impact of education on the EQ-5D-5L was nullified by having one or more chronic conditions.

Our second hypothesis was confirmed by the difference in patterns of EQ-5D scores in the predicted direction with respect to favourable occupational and insurance status, living situation, and level of disaster preparedness. As noted in Table 3, the magnitude of the difference of EQ-5D-5L index and LSS was larger for these factors than for race/ethnicity and income. Similar to education, the relative magnitude of these relationships for the EQ-5D-5L index and LSS differed according to if respondents were healthy or diseased. Lower scores for unemployed persons are consistent with Solomou & Constantinidou [32] who reported that unemployed persons had higher symptoms of depression and anxiety during COVID-19. In terms of living situation, respondents living with nonfamily roommates may experience a greater feeling of instability and susceptibility [33]. Additionally, the finding that lower levels of disaster preparedness were associated with worse HRQoL aligns with the work of Strine and colleagues [24]. The variation in EQ-5D-5L index and LSS between different categories of occupational status and loss of health insurance tended to be greater for diseased versus healthy respondents, especially for diseased respondents unable to work. Regarding possible explanations, employment and health insurance may serve as markers to access to care, and, as such, are more critical to persons with chronic diseases [18]. Therefore, HRQoL may be lower due to lack of access to medical care [34]. Of note, EQ VAS scores did not show the same patterns of having a more pronounced change with these factors or differing according to being healthy or diseased.

Our third hypotheses proved to be correct. Persons who reported smoking, chromic conditions, and recalling more difficulty with accessing healthcare had worse EQ-5D scores as compared to those who did not. Overall, these differences tended to have the greatest magnitude compared to individual characteristics (hypothesis 1) or occupational or living situation (except for being unable to work) (hypothesis 2), and diseased respondents who smoked and had worse access to care were more adversely impacted than healthy respondents (’amplification’). Scores on EQ outcomes declined with each additional chronic condition. This finding also has been noted by other investigators [27], and persons with two or more chronic conditions may avoid both urgent or emergency and routine medical care because of concerns over COVID-19 [35]. Similarly, EQ-5D scores tended to decline with worse experienced access to care, with EQ-5D-5L index and LSS showing a more marked decrease in diseased versus healthy respondents. These findings may indicate a more urgent need for accessible health care in the diseased respondents.

Our results that middle-aged and older persons tended to have higher EQ-5D-5L index and LSS compared to younger persons were unexpected and differ from the published literature [27, 36]. Such findings may be due to the disproportionate adverse effect of COVID-19 on younger persons. In terms of dimensions most affected, persons aged 18–24 reported not only more problems in anxiety/depression, but also in the other four dimensions. In their online survey of adults, Smith and colleagues [37] found that the youngest age group (18–24 year olds) had the worst mental health. During the time that our survey was administered, the frequency of depressive and anxiety symptoms in New York residents was 28.7% and 36.1%, respectively, with this percentage decreasing according to increasing age category [22]. This phenomenon may be related to response behaviour and response shift. Older respondents may respond in an age comparative manner, meaning responding relatively to others of a similar age, while younger respondents might experience and express concepts of HRQoL in a slightly different manner than older respondents [38]. We currently do not have a satisfactory explanation for the finding that this effect is even more pronounced in the diseased respondents. There are more than 30 population datasets in many countries of the world before COVID, with data from persons from the general population aged 18 years and older. None of these studies shows any indication of different understanding of the questions compared to elderly persons [13].

Understanding the general population HRQoL scores during COVID-19 is critical, given that the entire population is at risk for COVID-19 and different demographic subgroups will be affected in different ways. COVID-19 also has disrupted many segments of the economy, in addition to education and social relationships, and the chronic medical complications of COVID-19 are largely unknown [39]. Members of a given subgroup may experience different outcomes based on policies implemented at the local and state level. Capturing COVID-19’s effect in terms of both HRQoL (morbidity) and mortality is essential to estimating summary measures of population health such as quality-adjusted life years or disability-adjusted life years [40, 41].

Our investigation has several limitations. First, the data collection agency administered this survey only in English using an existing large Internet panel representative of the New York City and State (excluding NYC) populations. As a result, we cannot rule out selection bias, despite representativeness measures taken [42]. Second, while we combined the data from participants living in New York City (n = 1045) and New York State (n = 1612), these two participant groups may differ according to sociodemographic characteristics. Third, analyses were cross-sectional. Fourth, COVID-19-exposure and symptoms, risk factors for COVID-19 related complications, and chronic conditions were self-reported. Nevertheless, all condition-outcome relations satisfied clinical wisdom.

In conclusion, our results highlight the differential effect of a range of factors on HRQoL for New Yorkers when New York was the epicenter of the COVID-19 pandemic. Additional research should examine the relationship between mental health and scores of HRQoL among younger persons as well as how EQ-5D scores, and the specific dimensions affected, may change over time. Furthermore, as more and more New Yorkers have contracted COVID-19 and are experiencing long-term health effects, examining HRQoL over time amongst those patients will provide complementary information and, ultimately, enable the total burden of disease due to COVID-19 to be assessed.

## Supporting information

S1 Table Distribution of age, gender, level of education, and residency among dropouts and completers. (DOCX)

S2 Table Multivariable analysis of all respondents rescaled. (DOCX)

S3 Table Mean (SD) EQ-5D-5L index, level sum score, and EQ VAS scores by respondent’s characteristics. (DOCX)

S4 Table EQ-5D-5L, level sum score, and EQ VAS, univariable and multivariable analysis in healthy and diseased respondents. (DOCX)
