# Supplementary material for: Health inequities as measured by the EQ-5D-5L during COVID-19: Results from New York in healthy and diseased persons
Source: PLoS One. 2022 Jul 28;17(7):e0272252. doi: 10.1371/journal.pone.0272252 (PMC9333246; doi:10.1371/journal.pone.0272252)
Supplement: S1 Table — (DOCX) [file pone.0272252.s001.docx]

Supplementary File 1:

There are 364 people who started but did not complete the questionnaire (dropouts). We compared the distributions of age, gender, level of education, and residency between the respondents (completers) in our analysis and dropouts.

People who dropped out are significantly younger, mostly female.

# S1 Table: Distribution of age, gender, level of education, and residency among dropouts and completers.

|  | **Completers**  **(N=2657)** | **Dropouts**  **(N=364)** | **P value** |
| --- | --- | --- | --- |
| **Age** |  |  | < 0.001 |
| Median (IQR) | 48.0 (26.0) | 44.0 (25.0) |  |
| Mean (SD) | 47.6 (15.4) | 43.9 (15.8) |  |
| **Gender** |  |  | 0.043 |
| Male | 1193 (89.3%) | 143 (10.7%) |  |
| Female | 1464 (86.9%) | 221 (13.1%) |  |
| **Level of education** |  |  | < 0.001 |
| High | 2196 (88.5%) | 286 (11.5%) |  |
| Middle | 359 (88.0%) | 49 (12.0%) |  |
| Low | 102 (87.9%) | 14 (12.1%) |  |
| Unwilling to tell | 0 (0.0%) | 15 (100%) |  |
| **Residency** |  |  | 0.073 |
| NY City | 1045 (86.7%) | 161 (13.2%) |  |
| NY State | 1612 (88.8%) | 203 (11.2%) |  |
