# Supplementary material for: Health inequities as measured by the EQ-5D-5L during COVID-19: Results from New York in healthy and diseased persons
Source: PLoS One. 2022 Jul 28;17(7):e0272252. doi: 10.1371/journal.pone.0272252 (PMC9333246; doi:10.1371/journal.pone.0272252)
Supplement: S2 Table — (DOCX) [file pone.0272252.s002.docx]

Supplementary File 2:

We conducted a multivariable analysis of the EQ-5D-5L index, level sum score, and EQ VAS for all respondents based on specific health determinants. Positive coefficient indicates better health compared to the reference group. Statistical significance (0.05) is marked by a “*” on top of the coefficients.

EQ-5D-5L index and level sum score

Almost all factors relating to a lower social position showed a negative association (i.e., worse health), except for age, race/ethnicity, level of education, and household income. Older, non-Hispanic Asian middle-educated, and middle income had higher coefficients, respectively, compared to other levels in the same groups. Almost all factors showed a significant impact except for race/ethnicity, residency, job loss, essential worker (index) and health insurance (level sum score). All age groups and non-Hispanic Asian had a (significantly) positive impact. Infected with COVID-19, chronic conditions, and (very) bad access to health care had the worst impact.

EQ VAS

All the factors relating to a lower social position showed a negative association (i.e., worse health), except for job loss. Almost all factors showed significant impact except for race/ethnicity, residency, living situation, health insurance, smoking, and COVID-19 status. Job loss had a (significantly) positive impact. Chronic conditions and (very) bad access to health care had the worst impact.

Table 2. Multivariable analysis of all respondents rescaled

|  |  | $\boldsymbol{100\times}$  $\mathbf{Index}$ | $\mathbf{125-}$  $\boldsymbol{5\times LSS}$ | $\mathbf{VAS}$ |
| --- | --- | --- | --- | --- |
|  |  | Coef. | Coef. | Coef. |
| Intercept | | 97.4 | 97.7 | 92.4 |
| Age group | | * | * | ***** |
|  | 25-34 yrs. | 5.5 | 4.0 | -2.0 |
|  | 35-44 yrs. | 6.6 | 4.5 | -3.3 |
|  | 45-54 yrs. | 7.1 | 5.0 | -3.6 |
|  | 55-64 yrs. | 7.3 | 5.1 | -4.2 |
|  | 65-75 yrs. | 8.5 | 5.8 | -2.6 |
| Race/ethnicity | |  |  | ***** |
|  | Non-Hispanic black | 0.4 | 0.5 | -0.7 |
|  | Hispanic | 0.3 | 0.2 | -4.1 |
|  | Non-Hispanic Asian | 4.5 | 2.9 | -0.1 |
| Level of education | | ***** | ***** | ***** |
|  | High | -3.1 | -2.0 | 0.9 |
|  | Low | -8.9 | -5.3 | -6.7 |
| Household income | |  |  | ***** |
|  | Q5 – richest | -0.9 | -0.6 | 1.7 |
|  | Q4 – rich | -1.5 | -0.6 | 2.2 |
|  | Q2 – poor | -0.5 | -0.5 | -0.2 |
|  | Q1 – poorest | -2.8 | -1.7 | -2.3 |
|  | Unwilling to tell | 1.0 | 0.8 | 0.9 |
| Residency | |  |  |  |
|  | NY state | 0.6 | 0.5 | 0.0 |
| Occupational status | | ***** | ***** | ***** |
|  | Unemployed | -5.0 | -3.7 | -0.9 |
|  | Retired | -3.3 | -2.6 | -1.6 |
|  | Unable to work | -15.1 | -9.8 | -5.2 |
| Job loss due to COVID-19 | |  |  |  |
|  | Yes | -0.3 | -0.1 | 1.5 |
| Essential worker status | | * | ***** |  |
|  | Essential worker | -3.5 | -2.4 | 0.5 |
| Living situation | | ***** | ***** |  |
|  | Living with partner and/or family | -1.5 | -1.2 | 0.0 |
|  | Other | -7.2 | -4.9 | -1.8 |
| Health insurance | | ***** |  |  |
|  | No health insurance | -3.9 | -2.1 | 1.1 |
|  | Unknown | -6.4 | -3.6 | -2.2 |
| Loss of health insurance due to COVID-19 | | ***** | ***** | ***** |
|  | Yes | -12.9 | -8.9 | -3.6 |
| Disaster preparedness | | ***** | ***** | ***** |
|  | Somewhat well prepared | 0.5 | -0.1 | -2.4 |
|  | Somewhat prepared | -3.6 | -2.8 | -5.0 |
|  | Somewhat not prepared | -5.9 | -4.2 | -4.3 |
|  | Not prepared | -10.4 | -7.3 | -8.8 |
| Smoking status (including e-cigarettes) | | ***** | ***** |  |
|  | Some days | -6.9 | -5.2 | -1.8 |
|  | Every day | -5.1 | -3.3 | -0.2 |
| Number of chronic conditions | | ***** | ***** | ***** |
|  | 1 | -10.4 | -7.4 | -7.7 |
|  | 2 | -17.3 | -11.9 | -10.5 |
|  | 3 | -24.1 | -15.5 | -15.6 |
|  | 4 or more | -37.4 | -23.9 | -17.3 |
| Recall last healthcare visit, experience with access | | ***** | ***** | ***** |
|  | Good/Usually good | -2.2 | -1.8 | -4.0 |
|  | Fair/Sometimes good | -7.9 | -5.8 | -9.6 |
|  | Bad/Usually not good | -17.5 | -11.6 | -10.0 |
|  | Very bad/Never good | -23.0 | -13.6 | -10.0 |

Reference group: age 18-24, male, middle-educated, middle annual household income (75,000-99,999$), resides in NY City, employed, no job loss due to COVID-19, not essential worker, living alone, has health insurance, no insurance loss due to COVID-19, well prepared for disaster, non-smoker, not infected with COVID-19, no chronic conditions, experience with access is very good/always good.
