# Supplementary material for: Health inequities as measured by the EQ-5D-5L during COVID-19: Results from New York in healthy and diseased persons
Source: PLoS One. 2022 Jul 28;17(7):e0272252. doi: 10.1371/journal.pone.0272252 (PMC9333246; doi:10.1371/journal.pone.0272252)
Supplement: S3 Table — (DOCX) [file pone.0272252.s003.docx]

# S3 Table Mean (SD) EQ-5D-5L index, level sum score, and EQ VAS scores by respondent’s characteristics

|  |  | **EQ-5D-5L index** | **EQ-5D-5L level sum score** | **EQ VAS** |
| --- | --- | --- | --- | --- |
|  | **Characteristics** | Mean (SD) | Mean (SD) | Mean (SD) |
| **Total** | | 0.82 (0.26) | 7.5 (3.4) | 79.3 (17.4) |
| **Age groups** | | **Sig. between each two groups except for:**  **25 -34 yrs. – 35-44 yrs.,**  **35-44 yrs. – 45-54 yrs.,**  **45-54 yrs. – 55-64 yrs.,**  **55-64 yrs. – 65-75 yrs.** | | **Not sig.** |
|  | 18-24 yrs. | 0.67 (0.33) | 9.7 (4.3) | 77.2 (21.0) |
|  | 25-34 yrs. | 0.77 (0.30) | 8.2 (3.9) | 78.2 (19.2) |
|  | 35-44 yrs. | 0.80 (0.29) | 7.8 (3.8) | 79.3 (18.1) |
|  | 45-54 yrs. | 0.84 (0.25) | 7.3 (3.2) | 79.0 (16.5) |
|  | 55-64 yrs. | 0.87 (0.21) | 6.9 (2.7) | 79.7 (16.2) |
|  | 65-75 yrs. | 0.89 (0.16) | 6.7 (2.2) | 81.2 (15.1) |
| **Gender** | | **Not sig.** | | |
|  | Male | 0.82 (0.28) | 7.6 (3.7) | 79.4 (16.9) |
|  | Female | 0.82 (0.24) | 7.5 (3.2) | 79.3 (17.8) |
| **Race/ethnicity** | | **Sig. between each two groups except for:**  **White – Asian**  **Black – Hispanic** | | **Sig. between each two groups except for:**  **White – Asian**  **Black – Hispanic**  **Black – Asian** |
|  | Non-Hispanic White | 0.84 (0.24) | 7.3 (3.2) | 80.6 (16.0) |
|  | Non-Hispanic Black | 0.76 (0.31) | 8.3 (3.8) | 76.5 (18.5) |
|  | Hispanic | 0.72 (0.32) | 9.0 (4.2) | 72.7 (23.3) |
|  | Non-Hispanic Asian | 0.87 (0.22) | 6.9 (3.0) | 80.2 (15.9) |
| **Level of education^[[1]](#footnote-1)^** | | **Sig. between each two groups except for:**  **High-mid** | | **Sig. between each two groups** |
|  | High | 0.83 (0.26) | 7.5 (3.4) | 80.2 (16.4) |
|  | Middle | 0.83 (0.22) | 7.4 (2.9) | 77.4 (18.8) |
|  | Low | 0.66 (0.41) | 9.6 (5.1) | 67.4 (26.8) |
| **Household income** | | **Sig. between:**  **Q5-Q1**  **Q5-Q1**  **Q4-Q1**  **Q3-Q1**  **Q2-Q1** | | **Sig. between:**  **Q5-Q2**  **Q5-Q1**  **Q5-Q1**  **Q4-Q2**  **Q4-Q1**  **Q3-Q1**  **Q2-Q1** |
|  | Q5 – richest (≥150,000$) | 0.82 (0.29) | 7.5 (3.8) | 82.4 (15.1) |
|  | Q4 – rich (100,000-149,999$) | 0.85 (0.24) | 7.2 (3.2) | 82.7 (14.9) |
|  | Q3 – middle (75,000-99,999$) | 0.85 (0.23) | 7.3 (3.0) | 79.8 (15.0) |
|  | Q2 – poor (50,000-74,999$) | 0.84 (0.21) | 7.4 (2.8) | 79.1 (16.5) |
|  | Q1 – poorest (≤49,999$) | 0.76 (0.30) | 8.3 (3.9) | 73.8 (20.9) |
|  | Unwilling to tell | 0.88 (0.21) | 6.8 (2.8) | 81.2 (18.1) |
| **Neighborhood/residency** | | **Sig. between:**  **Manhattan – NY state**  **Bronx – NY state** | | **Not sig.** |
|  | Manhattan | 0.78 (0.30) | 8.1 (3.9) | 80.6 (18.4) |
|  | Staten Island | 0.83 (0.26) | 7.3 (3.4) | 78.7 (18.1) |
|  | Bronx | 0.74 (0.30) | 8.7 (4.0) | 75.2 (22.5) |
|  | Brooklyn | 0.81 (0.29) | 7.8 (3.8) | 77.8 (17.5) |
|  | Queens | 0.83 (0.25) | 7.4 (3.3) | 78.8 (17.7) |
|  | NY State | 0.84 (0.25) | 7.4 (3.2) | 79.6 (16.7) |
| **Occupational status** | | **Sig. between each two groups** | | **Sig. between each two groups except for:**  **Employed – retired** |
|  | Employed | 0.85 (0.24) | 7.1 (3.1) | 81.5 (15.8) |
|  | Unemployed**^[[2]](#footnote-2)^** | 0.73 (0.31) | 8.8 (4.0) | 75.9 (18.7) |
|  | Retired | 0.89 (0.17) | 6.7 (2.3) | 80.4 (16.5) |
|  | Unable to work | 0.62 (0.39) | 10.0 (4.9) | 67.8 (23.1) |
| **Job loss in household due to COVID-19** | | **Sig.** | | |
|  | No | 0.85 (0.24) | 7.2 (3.1) | 79.8 (16.7) |
|  | Yes | 0.74 (0.32) | 8.6 (4.1) | 77.7 (19.4) |
| **Essential worker status** | | **Sig.** | | **Not sig.** |
|  | Not essential worker | 0.84 (0.23) | 7.2 (3.1) | 79.2 (16.9) |
|  | Essential worker | 0.78 (0.31) | 8.2 (4.0) | 79.7 (18.5) |
| **Living situation** | | **Sig. between each two groups except for:**  **Alone – with partner and/or family** | | **Not sig.** |
|  | Living alone | 0.83 (0.27) | 7.3 (3.4) | 78.2 (18.7) |
|  | Living with partner and/or family | 0.82 (0.25) | 7.5 (3.3) | 79.9 (16.7) |
|  | Other | 0.73 (0.38) | 8.7 (4.9) | 74.9 (20.8) |
| **Health insurance** | | **Sig. between each two groups except for:**  **No – unknown** | | |
|  | Yes | 0.83 (0.25) | 7.4 (3.2) | 79.9 (16.7) |
|  | No | 0.70 (0.38) | 9.1 (4.8) | 74.7 (20.7) |
|  | Unknown | 0.71 (0.39) | 8.9 (4.9) | 72.8 (24.2) |
| **Loss of health insurance due to COVID-19** | | **Sig.** | | |
|  | No | 0.85 (0.23) | 7.2 (3.0) | 79.9 (16.7) |
|  | Yes | 0.59 (0.39) | 10.7 (4.9) | 73.9 (22.2) |
| **Disaster preparedness** | | **Sig. between each two groups except for:**  **Somewhat prepared - Somewhat not prepared**  **Somewhat prepared – not prepared**  **Somewhat not prepared – not prepared** | | |
|  | Well prepared | 0.84 (0.27) | 7.2 (3.5) | 82.7 (16.0) |
|  | Somewhat well prepared | 0.86 (0.18) | 7.1 (2.4) | 79.6 (15.7) |
|  | Somewhat prepared | 0.77 (0.30) | 8.3 (3.9) | 74.4 (19.3) |
|  | Somewhat not prepared | 0.70 (0.30) | 9.1 (3.9) | 72.2 (18.8) |
|  | Not prepared | 0.66 (0.40) | 9.8 (5.3) | 69.0 (24.3) |
| **Smoking status (incl. e-cigarettes)** | | **Sig. between each two groups except for:**  **Some days – every day** | | |
|  | Not at all | 0.87 (0.21) | 6.9 (2.8) | 80.5 (16.4) |
|  | Some days | 0.70 (0.30) | 9.3 (3.9) | 75.3 (21.3) |
|  | Every day | 0.72 (0.34) | 8.9 (4.4) | 77.3 (18.2) |
| **COVID-19 status** | | **Sig. between each two groups except for:**  **May be infected – infected but recovered** | | **Sig. between:**  **Not infected – may be infected** |
|  | Not infected | 0.87 (0.19) | 6.8 (2.6) | 81.0 (16.1) |
|  | May be infected | 0.66 (0.30) | 9.9 (4.0) | 71.7 (20.4) |
|  | Infected but recovered | 0.55 (0.45) | 10.9 (5.5) | 74.9 (23.7) |
|  | Infected and not recovered | 0.11 (0.58) | 16.1 (7.2) | 79.9 (19.6) |
| **Number of chronic conditions** | | **Sig. between each two groups except for:**  **2 – 3** | | **Sig. between each two groups except for:**  1 – 2  2 – 3  3 – 4 |
|  | 0 | 0.91 (0.18) | 6.4 (2.5) | 84.2 (13.9) |
|  | 1 | 0.76 (0.29) | 8.5 (3.8) | 75.1 (18.5) |
|  | 2 | 0.70 (0.29) | 9.3 (3.6) | 72.3 (19.2) |
|  | 3 | 0.61 (0.33) | 10.2 (3.8) | 67.6 (22.0) |
|  | 4 and more | 0.43 (0.36) | 12.5 (4.1) | 62.9 (19.2) |
| **Expected access to health care** | | **Not sig.** | | **Sig. between:**  **Level 1 – level 2**  **Level 1 – level 3** |
|  | Expect no difficulties to go | 0.82 (0.29) | 7.6 (3.7) | 81.7 (16.6) |
|  | Expect difficulties to go | 0.83 (0.23) | 7.5 (3.1) | 78.4 (16.6) |
|  | Don’t expect to go because I’m afraid of COVID-19 | 0.81 (0.26) | 7.6 (3.3) | 76.0 (19.1) |
|  | Don’t expect to go because I will not qualify to get appointments | 0.83 (0.29) | 7.4 (3.9) | 79.3 (19.4) |
| **Recall last healthcare visit, experience with access** | | **Sig. between each two groups except for:**  **Good – very bad**  **Fair –bad**  **Fair – very bad**  **Bad – very bad** | | |
|  | Very good/Always good | 0.86 (0.24) | 7.0 (3.1) | 83.1 (16.0) |
|  | Good/Usually good | 0.82 (0.23) | 7.6 (3.1) | 78.2 (16.2) |
|  | Fair/Sometimes good | 0.72 (0.31) | 9.1 (4.2) | 70.2 (19.8) |
|  | Bad/Usually not good | 0.57 (0.39) | 10.9 (4.9) | 66.8 (20.9) |
|  | Very bad/Never good | 0.49 (0.52) | 11.6 (6.4) | 67.5 (26.1) |

Education categories: High (ISCED 5 and above), Middle (ISCED 3-4), Low (ISCED 0-2). Details see methods.

2 Including caregiver and student

Between group variation is measured by pairwise t-test (that does not have the assumption of equal variance), significant terms (p value <0.05) are marked on top of each group.

1. [↑](#footnote-ref-1)
2. [↑](#footnote-ref-2)
