# Supplementary material for: Health inequities as measured by the EQ-5D-5L during COVID-19: Results from New York in healthy and diseased persons
Source: PLoS One. 2022 Jul 28;17(7):e0272252. doi: 10.1371/journal.pone.0272252 (PMC9333246; doi:10.1371/journal.pone.0272252)
Supplement: S4 Table — (DOCX) [file pone.0272252.s004.docx]

# S4 Table: EQ-5D-5L, level sum score, and EQ VAS, univariable and multivariable analysis in healthy and diseased respondents^[[1]](#footnote-1),^^[[2]](#footnote-2)^

|  |  | Univariable analysis | | | | Multivariable analysis for  Healthy respondents | | | Multivariable for  Diseased respondents | | |
| --- | --- | --- | --- | --- | --- | --- | --- | --- | --- | --- | --- |
|  |  | $\boldsymbol{100\times}$  $\mathbf{Index}$ | | $\mathbf{125-}$  $\boldsymbol{5\times LSS}$ | $\mathbf{VAS}$ | $\boldsymbol{100\times}$  $\mathbf{Index}$ | $\mathbf{125-}$  $\boldsymbol{5\times LSS}$ | $\mathbf{VAS}$ | $\boldsymbol{100\times}$  $\mathbf{Index}$ | $\mathbf{125-}$  $\boldsymbol{5\times LSS}$ | $\mathbf{VAS}$ |
|  | | Coef. | | Coef. | Coef. | Coef. | Coef. | Coef. | Coef. | Coef. | Coef. |
| Intercept | | / | | / | / | **95.0** | **96.1** | **89.8** | **91.5** | **93.8** | **91.7** |
| Age group | |  | |  |  |  |  |  |  |  |  |
|  | 25-34 yrs. | **10.4** | | **7.5** | 1.0 | 3.2 | **2.5** | **-3.7** | 4.8 | 3.7 | -1.3 |
|  | 35-44 yrs. | **13.8** | | **9.5** | 2.1 | **3.8** | **2.6** | **-3.9** | **7.0** | **5.0** | -3.6 |
|  | 45-54 yrs. | **17.4** | | **12.2** | 1.8 | 2.2 | 1.8 | **-4.9** | **9.2** | **6.4** | -3.4 |
|  | 55-64 yrs. | **20.2** | | **13.9** | 2.5 | 3.0 | 2.1 | **-5.0** | **9.6** | **6.8** | -4.5 |
|  | 65-75 yrs. | **22.5** | | **15.3** | **4.0** | 2.3 | 1.7 | **-5.0** | **12.3** | **8.4** | -1.2 |
| Gender | |  | |  |  |  |  |  |  |  |  |
|  | Female | 0.5 | | 0.1 | -0.1 | / | / | / | / | / | / |
| Race/ethnicity | |  | |  |  |  |  |  |  |  |  |
|  | Non-Hispanic Black | **-8.2** | | **-5.2** | **-4.1** | -0.2 | -0.3 | 1.4 | -1.0 | -0.1 | -2.7 |
|  | Hispanic | **-12.2** | | **-8.2** | **-7.9** | -2.4 | -1.3 | **-2.8** | 2.6 | 1.4 | **-5.5** |
|  | Non-Hispanic Asian | 3.1 | | 2.0 | -0.4 | **4.2** | **3.0** | 0.2 | 5.0 | 2.8 | -0.4 |
| Level of education | |  | |  |  |  |  |  |  |  |  |
|  | High | -0.6 | | -0.5 | **2.7** | -0.6 | -0.4 | **2.1** | -4.0 | -2.7 | 0.1 |
|  | Low | **-17.7** | | **-11.1** | **-10.0** | **-11.6** | **-7.2** | **-6.8** | -5.9 | -3.3 | **-6.3** |
| Household income | |  | |  |  |  |  |  |  |  |  |
|  | Q5 – Richest | -2.4 | | -1.2 | **2.6** | 0.8 | 0.7 | **2.4** | -2.1 | -1.3 | 1.7 |
|  | Q4 – Rich | 0.3 | | 0.2 | **2.9** | 0.3 | 0.3 | 1.4 | -3.3 | -1.7 | 1.9 |
|  | Q2 – Poor | -0.7 | | -0.6 | -0.7 | -0.5 | 0.1 | 1.6 | -1.4 | -1.6 | -2.7 |
|  | Q1 – Poorest | **-8.5** | | **-5.3** | **-6.0** | -1.3 | -0.7 | -1.0 | **-5.1** | **-3.2** | -3.8 |
|  | Unwilling to tell | 3.2 | | 2.4 | 1.4 | -0.2 | 0.1 | 1.5 | 0.7 | 0.7 | 0.3 |
| Residency | |  | |  |  |  |  |  |  |  |  |
|  | NY State | **3.4** | | **2.4** | 0.7 | 1.3 | 0.9 | 0.9 | 0.0 | 0.2 | -0.7 |
| Occupational status | |  | |  |  |  |  |  |  |  |  |
|  | Unemployed | **-12.3** | | **-8.6** | **-5.5** | -0.5 | -0.7 | -0.6 | **-7.0** | **-4.8** | -1.2 |
|  | Retired | **3.2** | | **1.9** | -1.1 | -0.3 | -0.5 | 1.3 | -5.6 | **-4.1** | **-5.0** |
|  | Unable to work | **-22.9** | | **-14.6** | **-13.7** | -3.1 | -1.9 | **-4.8** | **-21.4** | **-13.9** | **-5.8** |
| Job loss due to COVID-19 | |  | |  |  |  |  |  |  |  |  |
|  | Yes | **-10.3** | | **-6.9** | **-2.1** | 1.6 | 0.9 | **1.9** | -1.8 | -0.9 | 1.1 |
| Essential worker status | |  | |  |  |  |  |  |  |  |  |
|  | Essential worker | **-6.9** | | **-4.6** | 0.5 | -0.6 | -0.6 | 1.4 | **-4.4** | **-2.9** | -0.1 |
| Living situation | |  | |  |  |  |  |  |  |  |  |
|  | Living with partner and/or family | -1.1 | | -1.0 | **1.7** | -1.6 | -1.1 | 0.2 | -2.0 | -1.8 | -0.6 |
|  | Other | **-10.4** | | **-6.9** | -3.3 | **-8.3** | **-5.8** | -1.5 | **-8.5** | **-5.4** | -2.3 |
| Health insurance | |  | |  |  |  |  |  |  |  |  |
|  | No health insurance | **-12.8** | | **-8.2** | **-5.1** | -3.1 | -2.4 | -0.1 | -5.9 | -2.6 | 3.2 |
|  | Unknown | **-12.0** | | **-7.3** | **-7.1** | -1.9 | -1.5 | -2.7 | **-11.8** | **-6.3** | -1.3 |
| Loss of health insurance due to COVID-19 | |  | |  |  |  |  |  |  |  |  |
|  | Yes | **-26.1** | | **-17.7** | **-6.1** | -1.7 | -0.8 | -1.7 | **-12.5** | **-8.8** | **-3.3** |
| Disaster preparedness | |  | |  |  |  |  |  |  |  |  |
|  | Somewhat well prepared | -1.7 | | 0.7 | **-3.0** | -1.5 | **-1.3** | **-2.3** | 3.5 | 1.8 | -2.3 |
|  | Somewhat prepared | **- 7.7** | | **-5.6** | **-8.3** | **-2.5** | **-2.2** | **-3.8** | -2.0 | -1.8 | **-5.6** |
|  | Somewhat not prepared | **-14.1** | | **-9.6** | **-10.4** | -0.3 | -0.7 | -3.2 | -5.6 | **-3.9** | **-4.6** |
|  | Not prepared | **-18.8** | | **-12.8** | **-13.7** | **-6.6** | **-4.6** | **-11.6** | **-8.2** | **-6.0** | **-6.0** |
| Smoking status (including e-cigarettes) | |  | |  |  |  |  |  |  |  |  |
|  | Some days | **-16.7** | | **-11.9** | **-5.2** | **-3.7** | **-3.2** | 0.0 | **-7.5** | **-5.6** | -2.7 |
|  | Every day | | **-14.9** | **-9.8** | **-3.2** | -0.7 | -0.2 | -1.0 | **-6.3** | **-4.1** | 0.0 |
| Number of chronic conditions | | |  |  |  |  |  |  |  |  |  |
|  | 1 | | **-15.0** | **-10.5** | **-9.2** | / | / | / | -1.6 | -1.3 | **-3.3** |
|  | 2 | | **-21.3** | **-14.6** | **-12.0** | / | / | / | **-8.9** | **-6.1** | **-6.0** |
|  | 3 | | **-29.7** | **-19.3** | **-16.7** | / | / | / | **-14.7** | **-9.1** | **-10.9** |
|  | 4 and more | | **-47.9** | **-30.9** | **-21.3** | / | / | / | **-27.0** | **-16.9** | **-12.4** |
| Expected access to health care | | |  |  |  |  |  |  |  |  |  |
|  | Expect difficulties to go | 1.0 | | 0.1 | **-3.2** | / | / | / | / | / | / |
|  | Don’t expect to go be­cause I’m afraid of COVID-19 | - 0.3 | | -0.4 | **-5.6** | / | / | / | / | / | / |
|  | Don’t expect to go because I will not qualify to get appointments | 1.3 | | 0.6 | -2.4 | / | / | / | / | / | / |
| Recall last healthcare visit, experience with access | |  | |  |  |  |  |  |  |  |  |
|  | Good/Usually good | **- 4.2** | | -3.1 | **-4.9** | -1.4 | **-1.2** | **-3.0** | -2.6 | **-2.1** | **-4.8** |
|  | Fair/Sometimes good | **-14.9** | | **-10.6** | **-12.9** | **-4.9** | **-3.1** | **-7.8** | **-8.6** | **-6.7** | **-9.9** |
|  | Bad/Usually not good | **-29.9** | | **-19.9** | **-16.3** | -1.6 | -1.3 | -2.4 | **-22.7** | **-15.1** | **-12.9** |
|  | Very bad/Never good | **-37.1** | | **-23.0** | **-15.6** | -8.2 | -4.2 | **-15.6** | **-28.3** | **-16.8** | -5.4 |

Healthy respondents are those who reported no chronic conditions and not infected with COVID-19

2 Diseased respondents are those who reported one or more chronic condition(s) and/nor (possibly) infected with COVID-19

The outcomes variables are scaled: index was rescaled to 100 times index, level sum score (presented as LSS) was rescaled at 125 minus (5 times LSS).

Reference group in each analysis: age 18-24 yrs., male, middle-educated, middle annual household income ($75,000-99,999), resides in New York City, employed, no job loss due to COVID-19, not essential worker, living alone, has health insurance, no insurance loss due to COVID-19, well prepared for disaster, non-smoker, not infected with COVID-19, no chronic conditions, expected access to health care as used to, experience with access is very good/always good. Coefficients that reached significance level (p<0.05) were marked in bold.

1. [↑](#footnote-ref-1)
2. [↑](#footnote-ref-2)
